# The *5-HTTLPR*-rs25531 *S-A-S-A* Haplotype and Chronic Stress Moderate the Association Between Acute Stress and Internalizing Mental Disorders Among HIV+ Children and Adolescents in Uganda

**DOI:** 10.3389/fgene.2021.649055

**Published:** 2021-04-23

**Authors:** Allan Kalungi, Jacqueline S. Womersley, Eugene Kinyanda, Moses L. Joloba, Wilber Ssembajjwe, Rebecca N. Nsubuga, Soraya Seedat, Sian M. J. Hemmings

**Affiliations:** ^1^Department of Psychiatry, Stellenbosch University, Cape Town, South Africa; ^2^Mental Health Project, MRC/UVRI and LSHTM Uganda Research Unit, Entebbe, Uganda; ^3^Department of Psychiatry, Makerere University, Kampala, Uganda; ^4^Department of Medical Microbiology, Makerere University, Kampala, Uganda; ^5^South African Medical Research Council/Stellenbosch University Genomics of Brain Disorders Research Unit, Cape Town, South Africa; ^6^School of Biomedical Sciences, College of Health Sciences, Makerere University, Kampala, Uganda; ^7^MRC/UVRI and LSHTM Uganda Research Unit, Statistics and Data Science Section, Entebbe, Uganda

**Keywords:** internalizing mental disorders, acute stress, serotonin transporter gene, *5-HTTLPR*-rs25531, chronic stress, HIV+ children and adolescents, Uganda

## Abstract

**Background**: Internalizing mental disorders (IMDs) among HIV-positive (HIV+) children and adolescents are associated with poor disease outcomes, such as faster HIV disease progression. Although it has been suggested that the development of IMDs is moderated by interaction of stressful life events and vulnerability factors, the underlying etiology is largely unknown. Serotonin transporter gene [solute carrier family 6 member A4 (*SLC6A4*)] and human tryptophan hydroxylase 2 gene (*TPH2*) polymorphisms have been implicated in the development of IMDs. This study investigated the association between acute stress and IMDs, and moderation by chronic stress and genetic variants in *SLC6A4* and *TPH2*.

**Hypothesis**: Acute stress acts through genetic and environmental vulnerability factors to increase the risk of developing IMDs.

**Methods**: Polymorphisms in *SLC6A4* (*5-HTTLPR*, rs25531, *5-HTTLPR*-rs25531, and *STin2* VNTR) and *TPH2* (rs1843809, rs1386494, rs4570625, and rs34517220) were genotyped in 368 HIV+ children and adolescents (aged 5–17 years) with any internalizing mental disorder (depression, anxiety disorders, or posttraumatic stress disorder), and 368 age- and sex-matched controls, who were also HIV+. Chronic and acute stress categories were derived by hierarchical cluster analysis. Logistic regression analysis was used to assess the independent moderating effect of chronic stress and each selected polymorphism on the association between acute stress and IMDs.

**Results**: We observed a statistically significant association between severe acute stress and IMDs (*p* = 0.001). Children and adolescents who experienced severe acute stress were twice as likely to develop IMDs, compared to children and adolescents who experienced mild acute stress (*p* = 0.001). Chronic stress interacted with severe acute stress to increase the risk of IMDs (*p* = 0.033). Acute stress was found to interact with *5-HTTLPR*-rs25531 *S-A-S-A* haplotype to increase the risk for IMDs among Ugandan HIV+ children and adolescents (*p* = 0.049). We found no evidence for a combined interaction of acute stress, chronic stress, and *5-HTTLPR*-rs25531 on IMDs.

**Conclusion**: The odds of having an internalizing mental disorder (IMD) were higher among HIV+ children and adolescents who experienced severe acute stress compared to HIV+ children and adolescents who experienced mild acute stress. Chronic stress and *5-HTTLPR*-rs25531 independently moderated the association between acute stress and IMDs.

## Introduction

HIV-positive (HIV+) children and adolescents suffer a considerable burden of internalizing mental disorders (IMDs; Mellins et al., 2012; Nachman et al., 2012; Kinyanda et al., 2019). IMDs are characterized by quiet, internal distress (Tandon et al., 2011) and include depressive and anxiety disorders, as well as posttraumatic stress disorder (PTSD; American Psychiatric Association, 2013). IMDs among people living with HIV/AIDS have generally been associated negative outcomes of more rapid HIV disease progression (Ironson et al., 2005; Chida and Vedhara, 2009), poor adherence to medication (Springer et al., 2012; Kinyanda et al., 2018), risky sexual behavior (Springer et al., 2012; Kinyanda et al., 2018), and poor linkages to care (Bhatia et al., 2011).

Internalizing mental disorders are complex disorders with gene-environment interactions contributing to their etiology (Musci et al., 2019), where a number of genes play a role with each gene contributing a small effect (Plomin and Davis, 2009; Assary et al., 2018). The role of psychosocial factors and their interaction with biological mechanisms in the etiology of IMDs is still poorly understood. HIV+ children and adolescents experience various chronic life stressors such as awareness of their HIV-status, increased levels of stigma and poorer parental mental health (Betancourt et al., 2014). As chronic stressors are reported to be risk factors for IMDs (Adelman et al., 2014; Robles et al., 2014; Revenson et al., 2016), chronic stressors likely contribute to the etiology of IMDs among HIV+ children and adolescents (Boyes and Cluver, 2015; Lwidiko et al., 2018).

Internalizing mental disorders are heritable (Smoller, 2016): twin studies have estimated a genetic heritability of 35% for depression (Otte et al., 2016) and 30-50% for PTSD (Smoller, 2016). In addition, a meta-analysis of family and twin studies estimated a genetic heritability of 31.6% for generalized anxiety disorder (GAD; Hettema et al., 2001). A more recent genome-wide association study (GWAS) of monozygotic and dizygotic female twins has estimated a genetic heritability of 42% for GAD (Davies et al., 2015). Despite being heritable, the underlying etiology of IMDs is largely unknown although dysregulation in serotonergic transmission has been implicated in depression among adults living with HIV (Hammoud et al., 2010).

Serotonergic pathways in the central nervous system is important in regulating mood and anxiety (Olivier, 2015). After an impulse is fired and serotonin (5-HT) is released into the synaptic cleft, 5-HT re-uptake reduces the activity of the serotonergic neurons, preparing the neuron for a new discharge (Artigas, 2013a,b; Olivier, 2015). Encoded by the serotonin transporter gene [solute carrier family 6 member 4 (*SLC6A4*)], the serotonin transporter (5-HTT) influences serotonergic transmission by regulating the duration of serotonin in the synaptic cleft (Rudnick, 2006; Kristensen et al., 2011). 5-HTT is a target for selective serotonin re-uptake inhibitors (SSRIs) that competitively block substrate binding and thereby prolong neurotransmitter action at the synapse (Kristensen et al., 2011; Cipriani et al., 2018). Tryptophan hydroxylase 2 (TPH2) catalyzes the rate-limiting step in 5-HT biosynthesis (Walther et al., 2003; Carkaci-Salli et al., 2006) and is expressed exclusively in the brainstem (Kennedy et al., 2012), an area which is the major locus of serotonin-producing neurons (Kennedy et al., 2012). Long-term treatment with the SSRI fluoxetine has been found to be associated with concurrent upregulation of *TPH2* messenger ribonucleic acid (mRNA) expression and alleviation of depressive symptoms (Shishkina et al., 2007), suggesting that low *TPH2* gene expression could represent a dysregulation corrected by fluoxetine.

*SLC6A4* is located on chromosome 17 at position 17q11 (National Center for Biotechnology Information, 2021a). The serotonin transporter-linked polymorphic region (*5-HTTLPR*) within the promoter region of *SLC6A4* has been implicated in depression (Clarke et al., 2010; Wang et al., 2016), anxiety-related traits (Munafò et al., 2009b), and PTSD (Xie et al. 2009). *5-HTTLPR* variants comprise either 14 (short, *S*-allele) or 16 (long, *L*-allele) copies of a 22–23 base pair (bp) imperfect repeat (Heils et al., 1996). *In vitro* studies show that the *L*-allele has two to three times higher basal transcriptional activity compared to the *S*-allele (Lesch et al., 1996; Philibert et al., 2008). In proximity to *5-HTTLPR* is an *A* to *G* single nucleotide polymorphism (SNP), rs25531 which has been reported to alter expression of the *SLC6A4* by creating a functional AP2 transcription-factor binding site (Hu et al., 2006; Ehli et al., 2012). The rs25531 *G*-allele has been associated with reduced transporter gene expression (Hu et al., 2006; Ehli et al., 2012). This SNP, when analyzed in combination with *5-HTTLPR*, results in *L-A* and *L-G* haplotypes. The *L-G* haplotype has been associated with lower *SLC6A4* expression levels compared to the *L-A* haplotype (Hu et al., 2006; Lipsky et al., 2009). However, some studies have found no effect of the *L-G* haplotype on *SLC6A4* expression (Martin et al., 2007; Philibert et al., 2008).

A variable number of tandem repeats (VNTR) polymorphism also occurs within the second intron (*STin2*) of *SLC6A4*. The polymorphism consists of multiple repeat copies of a 16–17 bp element (Battersby et al., 1996; Furlong et al., 1998). Three alleles have been reported (National Center for Biotechnology Information, 2021b), containing 9 (*STin2.9*), 10 (*STin2.10*), and 12 (*STin2.12*) copies of the repeat. The alleles have been associated with differential expression of *SLC6A4*, as *STin2.9* has been found to be associated with increased *SLC6A4* expression, and an increasing number of repeats associated with reduced reporter gene expression in rat neonate prefrontal cortical cultures (Ali et al., 2010). The *STin2* VNTR polymorphism has also been found to interact with *5-HTTLPR* to regulate expression of *SLC6A4*, with the combination of the *5-HTTLPR S*-allele and either *STin2.10* or *STin2.12* associated with increased expression, as compared to *STin2.9* whose combination with *5-HTTLPR S*-allele directed expression levels comparable to the *5-HTTLPR S*-allele alone (Ali et al., 2010; Haddley et al., 2012). The *STin2.9* allele has been associated with anxiety in patients with self-harming behaviors (Evans et al., 2013), *STin2.10* has been associated with both anxiety and PTSD (Evans, Li and Whipple, 2013; Xiao et al., 2019) and the *STin2.12* allele has been associated with depression, neuroticism, and suicide (Lopez de Lara et al., 2007; O’Gara et al., 2008; Kalungi et al., 2017).

The TPH2 gene is located on chromosome 12 at position 12q21.1. Located within intron 5 (rs1843809 and rs1386494), and within the promoter region (rs4570625), rs1843809, rs1386494, and rs4570625 have been linked to depression (Zill et al., 2004; Anttila et al., 2009; Gao et al., 2012). Located within the *TPH2* transcription factor binding site, rs34517220 SNP has been reported to modulate *TPH2* expression by altering binding sites for *foxa1* and *foxa2* transcription factors (Pristerà et al., 2015). Also, *foxa2* plays a role in establishing progenitor domains for serotonergic neuron precursors in the ventral hindbrain and in activating transcription factors required for the terminal differentiation of serotonergic neurons (Jacob et al., 2007). Therefore, rs34517220-driven variation in *foxa2* binding may have important effects on the development of neural serotonergic systems.

**Figure 1:**
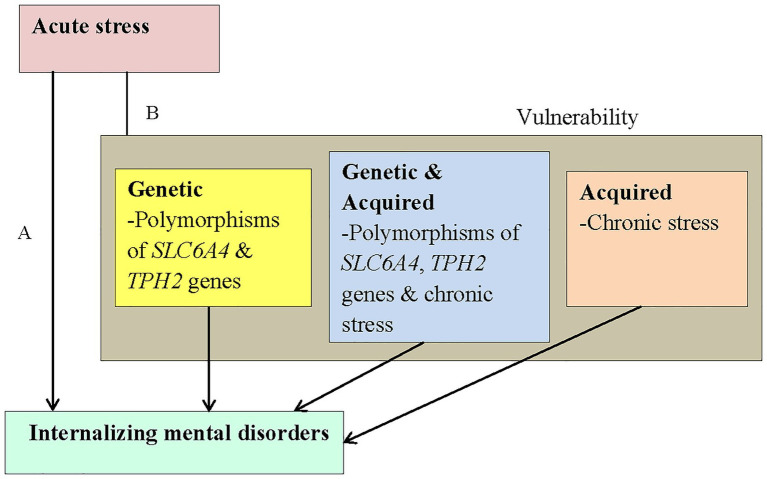
The conceptual framework is adapted from the diathesis-stress model for depression (Monroe and Simons, 1991). The aim of the present study was to investigate the association between acute stress and IMDs **(A)**. We postulated that acute stress would interact with vulnerability factors [**B**; genetic (yellow block), acquired (pink block), or both (blue block)] to increase the risk for IMDs. SLC6A4, serotonin transporter gene; TPH2, tryptophan hydroxylase 2 gene.

The diathesis-stress hypothesis of neuropsychiatric disorders postulates that a lower stress threshold is required for psychiatric disease to occur in individuals who harbor certain vulnerability factors, which may be genetic and/or acquired (Monroe and Simons, 1991; Silberg et al., 2001; Caspi et al., 2003; Figure 1). Stress is a common environmental risk factor for a number of mental disorders, including depression, anxiety, and PTSD (de Kloet et al., 2005; Popoli et al., 2011), and it is currently accepted that gene-environment interactions underlie the etiology of many, if not all, IMDs (Caspi and Moffitt, 2006; Willis and Brock, 2018; Jawahar et al., 2019).

In the context of the diathesis-stress hypothesis, with acute stress representing the exposure variable in a sample of HIV+ children and adolescents, we investigated polymorphisms in *SLC6A4* (*5-HTTLPR*, rs25531, and *STin2* VNTR) and *TPH2* (rs1843809, rs1386494, rs4570625, and rs34517220) as genetic vulnerability factors and chronic stress as an acquired vulnerability factor for IMDs. Our *a priori* selection of these polymorphisms was based on their associations with IMDs (*5-HTTLPR*, *STin2* VNTR, *TPH2* rs1843809, rs1386494, rs4570625, and rs34517220) and regulation of *SLC6A4* (*STin2* VNTR, rs25531).

## Materials and Methods

### Study Design

This case-control study was nested within a study which investigated mental health among HIV infected children and adolescents in Kampala and Masaka, Uganda (CHAKA study). Characteristics of the study participants and the study design for the CHAKA study are detailed elsewhere (Mpango et al., 2017; Kinyanda et al., 2019).

All study participants were of Bagandan ethnicity, the largest ethnic group in Uganda, and were thus expected to be genetically similar since relatively modest genetic differentiation has been observed among populations representing the major sub-populations in sub-Saharan Africa (Gurdasani et al., 2015). All participants in the present study were HIV+ children and adolescents (aged 5–17 years) of black African (Ugandan) ancestry (Kinyanda et al., 2019). Cases (*n* = 368) were defined as participants who had any depressive disorder [depression or dysthymia (persistent depressive disorder)] or any anxiety disorder (GAD, separation anxiety disorder, social anxiety disorder, panic disorder, and agoraphobia) or PTSD. Controls (*n* = 368) were age-, site-, socio-economic status (SES)-, and sex-matched without any psychiatric disorder. Genomic DNA was extracted from an archived cell pellet sample for each participant.

### Clinical Assessments

Children and assented adolescents, as well as their caregivers, were interviewed using a structured questionnaire. The questionnaire included, among others, socio-demographic characteristics (sex, study site, age, caregiver level of education, and SES) and modules on different psychiatric disorders from the DSM-5 referenced Children and Adolescent Symptom Inventory 5 (CASI-5; caregiver reported; Gadow and Sprafkin, 2013) and the Youth Inventory-4R (YI-4R; youth reported; Gadow and Sprafkin, 1999). The CASI-5 and YI-4R list the symptoms of a wide range of psychiatric disorders including major depressive disorder, GAD, PTSD, and attention-deficit/hyperactivity disorder, among others. Individual CASI-5 items are rated on a 4-point frequency of occurrence scale ranging from never (0) to very often (3). Though there are several CASI-5 scoring algorithms, in the present study, we used symptom count cut-off scores that reflect the prerequisite number of symptoms for a clinical diagnosis. About 4 ml of blood was withdrawn from each study participant by venipuncture into an EDTA vacutainer and was stored at −80°C pending DNA extraction for the genetics analyses.

### Inclusion and Exclusion Criteria

Inclusion criteria were: (i) HIV-infected outpatients, registered with an HIV clinic at any of the study sites; (ii) aged between 5 and 17 years at the time of enrolment; (iii) conversant in English or Luganda, the language into which research assessment tools were translated; and (iv) able to provide written informed consent (caregiver) and assent (adolescents). Exclusion criteria were: (i) seriously ill and (ii) being unable to understand study procedures.

### Ethical Considerations

The study was conducted in compliance with the Code of Ethics of the World Medical Association (Declaration of Helsinki). The CHAKA study obtained ethical and scientific clearance from the Uganda Virus Research Institute’s Science and Ethical Committee (# GC/127/15/06/459) and the Uganda National Council of Science and Technology (# HS 1601). The study reported here obtained approval from the Higher Degrees Research and Ethics Committee of the School of Biomedical Sciences, Makerere University (# SBS 421) and the Health Research Ethics Committee of Stellenbosch University (# S17/09/179). All caregivers provided informed consent for their children/adolescents to participate in the study and for a blood specimen to be drawn from them (child/adolescent) for genetics analyses. Adolescents further provided informed assent to participate in the study. Study participants who were diagnosed with significant psychiatric problems were referred to mental health units at Entebbe and Masaka government hospitals.

### Selection of Cases and Controls

The procedure for selection of cases and controls has been previously described (Kalungi et al., 2021). Briefly, all cases were stratified by site, sex, age category, and SES, resulting in a total of 36 strata. In each stratum, the number of cases was ascertained (e.g., for males in site 1 in the youngest age category and the lowest SES group there were nine cases). An equal number of controls were then randomly sampled from the stratum concerned, thus the controls were matched to the cases by site, sex, age, and SES.

### DNA Extraction

DNA was extracted from whole blood using the QiAmp Mini DNA Extraction Kit according to manufacturer’s instructions (Qiagen GmbH, Germany).

### Selection of Variants Within the Serotonin Transporter and Tryptophan Hydroxylase 2 Genes

The selection of variants was based on their role in regulating gene expression or association with any of the IMDs. Also, because the Bantu group of people in East Africa have been reported to genetically cluster together as per principal components analysis (unpublished results from NeuroGAP data), a minor allele frequency cut-off of at least 0.1 was set for each selected SNP based on the Luhya, a population that belongs to same Bantu group as the population of the present study. The minor allele frequency data were obtained from an online genomic project (Clarke et al., 2017). Supplementary Table S1 summarizes the biological data based on for selection for each of the *SLC6A4* and *TPH2* variants.

### *SLC6A4* Genotyping

All polymerase chain reactions (PCR) for *SLC6A4* polymorphisms were performed in a GeneAmp PCR System 9700 (Perkin Elmer Biosystems, Foster City, CA, United States). Amplification reactions were carried out in 25 μl reaction volumes containing: DNA template, 200 μM dNTP (Kapa Biosystems, Cape Town, South Africa), 5 μl of 10X Taq DNA polymerase buffer (Kapa Biosystems), 1.0 mM magnesium chloride (Kapa Biosystems), 0.625 units (U) Taq DNA polymerase (Kapa Biosystems), and 0.5 μM of each primer (Integrated DNA Technologies, Coralville, IA, United States), with bi-distilled water.

The *5-HTTLPR*, *5-HTTLPR*-rs25531, and *STin2* VNTR polymorphisms were genotyped following a procedure described by Kalungi et al. (2017). Fragment sizes revealed by PCR were confirmed by restriction fragment length analysis on the ABI prism. Expected fragment sizes of the alleles at the *5-HTTLPR*-rs25531 locus were as follows: *S-A* = 281 bp, *L-A* = 325 bp and *S-G-L-G* = 151 bp, resulting into the following genotypes: *S-A-S-A* = 281 bp; *L-A-L-A* = 325 bp; *S-G-S-G*, *L-G-L-G*, *L-G-S-G* = 151 bp; *L-G-S-A* = 151 + 281 bp and *L-A-S-G, L-A-L-G* = 325 + 151 bp. Expected fragment sizes of the alleles at the STin2 VNTR locus were as follows: 9-repeat (*STin2.9*) = 250 bp, 10-repeat (*STin2.10*) = 265 bp and 12-repeat (*STin2.12*) = 300 bp, resulting in the following genotypes: 9/9 = 250 bp, 9/10 = 250 + 265 bp, 9/12 = 250 + 300 bp, 10/10 = 265 bp, 10/12 = 265 + 300 bp, and 12/12 = 300 bp.

### Determining Genotypes for Selected Single Nucleotide Polymorphims in Tryptophan Hydroxylase 2 Gene

DNA samples were quantified and sent to LGC laboratory (LGC, Middlesex, United Kingdom) for automated SNP genotyping using the kompetitive allele-specific PCR (KASP) assay. Genotypes were determined for *TPH2* rs1843809, rs1386494, rs4570625, and rs34517220. However, none of these genotypes were validated using an alternate method.

### Haplotype Analyses

We used Haploview version 4.2 (Barrett, 2009) to analyze linkage disequillibrium (LD) in *SLC6A4* and *TPH2*. *SLC6A4* analyses included 5-*HTTLPR*, rs25531, and *STin2* VNTR polymorphisms. *5-HTTLPR* and *STin2* VNTR alleles were coded as dummy variables, i.e., *S* = 1, *L* = 2, and *STin2.10* = 1 and *STin2.12* = 2. As very few participants carried the *STin2.9* allele (*n* = 6), they were excluded from the analysis.

*TPH2* analyses included the three single nucleotide polymorphisms (SNPs) genotyped. Participants with more than 50% missing genotypes were excluded from the analysis, yielding data from 692 participants for each of the *SLC6A4* and *TPH2* analyses. None of the investigated *TPH2* polymorphisms were in LD (D’ < 0.8), while *SLC6A4 5-HTTLPR* and rs25531 were in LD (D’ > 0.8) (Supplementary Figures S1, S2).

### Generation of Acute and Chronic Stress Class Categories

Social disadvantage variables were grouped into an index of acute and chronic stress. Caregiver mental state (assessed as psychological distress using the Self-report Questionnaire-20; Beusenberg et al., 1994), child-caregiver relationship [assessed as child-caregiver interactions, using data on how often the caregiver (i) beats, (ii) insults, (iii) spanks, or (iv) yells at the child/adolescent] and HIV symptoms were grouped together to constitute “acute stress” and orphanhood, study site (urban vs. rural), and caregiver level of education as variables constituting “chronic stress.” Variables were scored on a disadvantage scale where, for example, double orphanhood carried a higher chronic stress score vs. single orphanhood or not orphaned; food availability: not enough food carried a higher chronic stress score vs. enough food; study site: urban carried a higher chronic stress score than rural; and caregiver level of education: no formal education carried a higher chronic stress scores than primary and primary a higher stress score than secondary, etc. Hierarchical cluster analysis using Statistica 13.5 software (TIBCO, CA, United States), Euclidian distance, as distance measure and Ward’s method for clustering (Ward, 1963), was used to generate the different cut-off points for each acute and chronic stress class, respectively.

The acute stress index ranged from 0 to 2.46, with a normal distribution, while the chronic stress index ranged from 0 to 3.75, with a normal distribution as well. A total of three classes were generated for each type of stress by the hierarchical cluster analysis, i.e., mild, moderate, and severe. For acute stress, the mild class had an acute stress score of less than 0.362, the moderate class a score of 0.362–0.622, while the severe class a score of greater than 0.622. For chronic stress, the mild class had a chronic stress score of less than 1.375, the moderate class a score of 1.375–2.375, while the severe class a score of greater than 2.375.

### Power of the Study

Using Stata 15 (StataCorp, TX, United States) software, we did a *post hoc* power calculation. Given a case-control ratio of 1:1, and assuming a zero correlation of exposure between cases and controls, a probability of 0.5 of exposure among controls at a 0.05 significance level, and an expected odds ratio of at least 1.8 for IMDs under severe acute stress, we needed 188 cases to achieve a power of 80% (Lachin, 1992). Our study comprising 368 cases was, therefore, adequately powered to address the main aim. Assuming similar assumptions, our interaction analyses achieved a power of 87.5%.

### Statistical Methods

Statistical analyses were conducted using Stata 15 (StataCorp, TX, United States). Socio-demographic characteristics were compared between cases and controls. SES was generated from a scale of nine household items owned (car, motorcycle, refrigerator, electricity, bicycle, radio, telephone, cupboard, and flask) as previously described (Kalungi et al., 2019). A *t*-test was used to compare the distribution of cluster of differentiation 4 (CD4) counts between cases and controls. We computed 95% confidence intervals and statistical significance was set at a value of *p* less than or equal to 0.05.

Genotype distributions were compared between cases and controls. Likelihood-ratio tests were used to test genotypes for the Hardy-Weinberg equilibrium (HWE) in both cases and controls. Logistic regression was used to assess the relationship between *TPH2* rs1843809 and IMDs (equation for the model was Y = β_0_ + β_1_X_1_, where Y = IMDs, β_0_ = constant, X_1_ = rs1843809, and β1 = coefficient for rs1843809). Logistic regression models were used to assess the relationship between acute stress and IMDs (equation for the model was Y = β_0_ + β_1_X_1_, where Y = IMDs, β_0_ = constant, X_1_ = acute stress, and β1 = coefficient for acute stress).

The moderating effect of chronic stress on the association between acute stress and IMDs was assessed by comparing logistic regression models of the association between acute stress and IMDs with and without chronic stress. Interactions between acute stress and chronic stress were tested using a likelihood ratio test (equation for the model was Y = β_0_ + β_1_X_1_ + β_2_X_2_ + β_3_X_1_X_2_, where Y = IMDs, β_0_ = constant, X_1_ = acute stress, X_2_ = chronic stress and β_1_–β_3_ are the coefficients for acute stress, chronic stress, and the interaction term, respectively).

The moderating effect of each genotype on the association between acute stress and IMDs was assessed by comparing logistic regression models of the association between acute stress and IMDs with and without the polymorphism controlling for chronic stress. Interactions between acute stress and each of the polymorphism were tested using a likelihood ratio test, controlling for chronic stress (equation for the model was Y = β_0_ + β_1_X_1_ + β_2_X_2_ + β_3_X_3_ + β_4_X_1_X_2_, where Y = IMDs, β_0_ = constant, X_1_ = acute stress, X_2_ = polymorphism/SNP, X_3_ = chronic stress and β_1_–β_4_ are the coefficients for acute stress, the polymorphism/SNP, chronic stress, and the interaction, respectively). These interaction models were performed on all the explanatory variables even without observing significant main effects, in order to rule out the possibility of cross-over interaction, where significant interactions may be observed for non-significant main effects (Widaman et al., 2012). Three-way interactions of acute stress, chronic stress, and any selected polymorphism on IMDs were not assessed as the two-way models were better than the three-way models, based on goodness of fit test, Arkaike informatics criteria, and the Bayesian information criterion. The 95% confidence intervals were calculated for all analyses.

## Results

Socio-demographic factors were similarly distributed between cases and controls as shown in Table 1.

**Table 1:** Distribution of socio-demographic factors in cases and controls.

Variable (*n*, %)	Case *n* (%)	Control *n* (%)	*p*
Sex			0.111
Male (342, 46.5%)	160 (43.6)	182 (49.5)	
Female (393, 53.5%)	207 (56.4)	186 (50.5)	
Site			0.941
Urban (415, 56.4%)	208 (56.5)	207 (56.3)	
Rural (321, 43.6%)	160 (43.5)	161 (43.7)	
Age			0.374
7-11 years (389, 55.9%)	202 (57.6)	187 (54.2)	
12-17 years (307, 44.1%)	149 (42.4)	158 (45.8)	
Education level			0.371
No formal education (13, 1.8%)	9 (2.5)	4 (1.1)	
Primary (648, 88.4%)	323 (88.0)	325 (88.8)	
Secondary (72, 9.8%)	35 (9.5)	37 (10.1)	
Socioeconomic status			0.459
Low (332, 45.1%)	171 (46.5)	161 (43.8)	
High (404, 54.9%)	197 (53.5)	207 (56.2)	
Mean CD4 count at baseline	947.04	944.02	0.939

CD4, cluster of differentiation 4; primary, 0–7 years of formal education; Secondary, 8–14 years of formal education; Low socioeconomic status, 0–13; High socioeconomic status, >13. All numbers that do not add up to the total of 736 participants were due to missing data.

The HWE *p*-values among cases and controls for each polymorphism are shown in Table 2. None of the genotype frequencies deviated significantly from HWE among controls except for rs1386494, which was thus omitted from further analysis. For *SLC6A4* polymorphisms, *5-HTTLPR* was in LD with rs25531 (D’ > 0.8) (Supplementary Figure S1), while for *TPH2* SNPs, none of the selected polymorphisms were in LD (D’ < 0.8) (Supplementary Figure S2).

**Table 2:** Distribution of genotypes and Hardy-Weinberg equilibrium analysis between cases and controls for *SLC6A4* [*5-HTTLPR*, rs25531, *5-HTTLPR*-rs25531, and *STin2* VNTR] and *TPH2* [rs1843809, rs1386494, rs34517220, and rs4570625] polymorphisms.

Genotype	Cases (*n* = 368) *n* (%)	Controls (*n* = 368) *n* (%)	p^*^	HWE Cases	HWE Controls
*5-HTTLPR* (*n* = 698)			0.286	0.323	0.1916
*LL*	223 (62.3)	231 (67.9)			
*LS*	115 (32.1)	94 (27.7)			
*SS*	20 (5.6)	15 (4.4)			
rs25531 (*n* = 685)			0.988	0.795	0.641
*AA*	227 (65.4)	221 (65.4)			
*AG*	108 (31.1)	106 (31.4)			
*GG*	12 (3.5)	11 (3.2)			
*5-HTTLPR*-rs25531 haplotype (*n* = 685)			0.688	N/A	N/A
*L-A-L-A*	130 (37.4)	136 (40.2)			
*L-A-L-G*	76 (21.9)	82 (24.3)			
*L-A-S-A*	79 (22.8)	71 (21.0)			
*L-A-S-G*	2 (0.6)	Absent			
*L-G-L-G*	12 (3.5)	11 (3.3)			
*S-A-S-A*	18 (5.2)	15 (4.4)			
*S-A-L-G*	30 (8.6)	23 (6.8)			
*STin2* VNTR (*n* = 687)			0.203	0.090	0.440
10/10	26 (7.5)	28 (8.8)			
10/12	126 (36.3)	112 (35.3)			
12/12	189 (54.5)	177 (55.9)			
9/9	5 (1.4)	Absent			
9/12	1 (0.3)	Absent			
rs1843809 (*n* = 686)			0.003	0.009	0.537
*TT*	105 (30.1)	113 (33.5)			
*TG*	150 (43.0)	169 (50.2)			
*GG*	94 (26.9)	55 (16.3)			
rs1386494 (*n* = 685)			0.06	0.956	0.007
*GG*	210 (59.7)	202 (60.7)			
*GA*	124 (35.2)	125 (37.5)			
*AA*	18 (5.1)	6 (1.8)			
rs34517220 (*n* = 685)			0.945	0.557	0.913
*AA*	90 (25.6)	84 (25.1)			
*AG*	170 (48.4)	166 (49.7)			
*GG*	91 (25.9)	84 (25.2)			
rs4570625 (*n* = 683)			0.574	0.787	0.229
*GG*	88 (25.2)	90 (26.9)			
*GT*	177 (50.7)	156 (46.7)			
*TT*	84 (24.1)	88 (26.4)			

5-HTTLPR, serotonin transporter linked polymorphic region; STin2 VNTR, serotonin transporter intron 2 variable number of tandem repeats; HWE, Hardy-Weinberg equilibrium; N/A, not applicable.

* Value of *p* for association of each investigated polymorphism with IMDs.

The association between each investigated polymorphism and IMDs is shown in Table 2. There was a protective effect against IMDs in participants with *TPH2* rs1843809 *TG* and *TT* genotypes compared with participants with the *GG* genotype (Table 3).

**Table 3:** Association between tryptophan hydroxylase 2 gene rs1843809 and internalizing mental disorders (IMDs).

IMDs	Odds ratio	*p* > |Z|	95% confidence interval
rs1843809 *GG*	Reference		
rs1843809 *TG*	0.52	0.001	0.349–0.774
rs1843809 *TT*	0.54	0.005	0.355–0.832

IMDs, internalizing mental disorders.

Table 4 shows the association between acute stress and IMDs. A significant association was observed between acute stress and being an IMD case. Table 5 shows the independent effect of chronic stress and *SLC6A4 5-HTTLPR*-rs25531 haplotype on the association seen in Table 4 (between acute stress and IMDs). The independent effect of all other polymorphisms on the association between acute stress and IMDs are shown in Supplementary Table S2.

**Table 4:** Association between acute stress and IMDs.

Acute stress class	OR (IMDs)	*p* > |Z|	95% CI	*p*
Mild	Reference			
Moderate	1.1	0.687	0.758–1.523	0.001
Severe	1.9	0.001	1.298–2.651	

OR, odds ratio; IMDs, internalizing mental disorders; CI, confidence interval.

**Table 5:** Logistic regression analyses for the interaction of acute stress with *5-HTTLPR*-rs25531 and chronic stress, respectively, on IMDs.

Model	Variable	Odds ratio	*p* > |Z|	95% CI	*p*
Excluding any polymorphism or chronic stress	Mild acute stress	Reference			0.046^a^
	Moderate acute stress	1.22	0.282	0.848–1.761	
	Severe acute stress	1.92	0.001	1.327–2.785	
Including 5-*HTTLPR-*rs25531	Acute stress*5-HTTLPR-**rs25531**				
	Mild AS*L-A-S-G	1			0.049^b^
	Moderate AS*L-A-L-G	2.52	0.022	1.142–5.540	
	Moderate AS*L-A-S-A	1.60	0.235	0.736–3.497	
	Moderate AS*L-A-S-G	0.63	0.124	0.351–1.135	
	Moderate AS*L-G-L-G	0.83	0.870	0.092–7.535	
	Moderate AS*S-A-L-G	1.48	0.563	0.395–5.500	
	Moderate AS*S-A-S-A	14.83	0.030	1.294–170.010	
			
	Severe AS*L-A-L-G	3.72	0.002	1.630–8.474	
	Severe AS*L-A-S-A	2.82	0.013	1.248–6.350	
	Severe AS*L-A-S-G	1.16	0.629	0.641–2.091	
	Severe AS*L-G-L-G	0.85	0.872	0.121–6.010	
	Severe AS*S-A-L-G	1.58	0.493	0.426–5.881	
	Severe AS*S-A-S-A	11.95	0.011	1.755–81.331	
Including chronic stress	Acute stress*Chronic stress				
	Moderate AS*moderate CS	1.04	0.896	0.607–1.768	0.033^c^
	Moderate AS*severe CS	1.80	0.035	1.041–3.106	
				
	Severe AS*moderate CS	0.95	0.904	0.406–2.217	
	Severe AS*severe CS	4.28	0.001	1.832–10.012	

AS, acute stress; CS, chronic stress; Acute stress*5-HTTLPR-rs25531, interaction between acute stress and 5-HTTLPR-rs25531 genotypes on internalizing mental disorder (IMDs).

a Value of *p* for association between acute stress and IMDs (the model without chronic stress/SLC6A4).

b Value of *p* for the likelihood-ratio test of interaction between acute stress and 5-HTTLPR-rs25531 on IMDs.

c Value of *p* for the likelihood-ratio test of interaction between acute stress and chronic stress on IMDs.

Apart from the *SLC6A4 5-HTTLPR*-rs25531 haplotype (Table 5), we found no significant interactions between acute stress and any polymorphism on IMDs (Supplementary Table S2). A significant interaction between acute and chronic stress (*p* = 0.033) and acute stress and *5-HTTLPR*-rs25531 *S-A-S-A* haplotype (*p* = 0.049) was observed on IMDs (Table 5). The odds of having an IMD were 4.3 times higher among participants under both severe acute stress and severe chronic stress, compared to those under mild acute stress and mild chronic stress (Table 5). The odds for being a case of an IMD were 14.8 times higher in participants with the *S-A-S-A* haplotype who experienced acute stress as compared to participants with the *L-A-L-A* haplotype who experienced mild acute stress. Similarly, the odds of having an IMD were 11.95 times higher in *S-A-S-A* participants with severe acute stress compared to *L-A-L-A* participants with mild stress (Table 5).

## Discussion

This study investigated the association between acute stress and IMDs, and whether this association was moderated by chronic stress or selected genetic variants in *SLC6A4* and *TPH2*. To our knowledge, this is the first sub-Saharan African study to investigate these interactions among HIV+ children and adolescents. Results revealed a statistically significant association between acute stress and IMDs. This association was found to be moderated by *5-HTTLPR*-rs25531 in a haplotype-dependent manner. Specifically, in comparison to *L-A-L-A* haplotype carriers with experience of mild acute stress, individuals carrying the *S-A-S-A* haplotypes were more likely to have an IMD under conditions of moderate and severe acute stress. We found no evidence for a significant combined interaction of acute stress, chronic stress, and *5-HTTLPR*-rs25531 on IMDs.

Stress represents a prevalent environmental risk factor for many mental disorders, including depression and anxiety (de Kloet et al., 2005; Popoli et al., 2011). Acute stress has been associated with the likelihood of developing IMDs among samples of adolescent school children and adult disaster workers (Fullerton et al., 2004; O’Connor et al., 2010; Brown et al., 2016). In line with previous studies, where acute stress has been associated with IMDs such as depression, PTSD (Fullerton et al., 2004), and anxiety (Grillon et al., 2007), we found that risk for IMDs increased with increasing acute stress and was highest for severe acute stress. Chronic stress was found to significantly moderate the association between acute stress and IMDs. Previous studies have reported on chronic stress as a risk factor for IMDs (Charney and Manji, 2004; de Kloet et al., 2005; Evans et al., 2013; Adelman et al., 2014; Robles et al., 2014; Revenson et al., 2016). Vulnerability to IMDs by chronic stress (through an interaction with acute stress) could be due to alterations in the functioning of the hypothalamic-pituitary-adrenal axis that follow exposure to chronic stress (de Kloet et al., 2005; Fuchs and Flïugge, 2006). In addition, chronic stress has been reported to affect serotonergic signaling in the brain (van den Buuse and Hale, 2019). As serotonergic systems have been implicated in threat appraisal, psychophysiological measures of stress response (skin conductance and startle reactions), and attentional bias to negative stimuli, it is possible that alterations in 5-HT signaling due to chronic stress may at least partially contribute to some of the behavioral features of IMDs (Pergamin-Hight et al., 2012; Klumpp et al., 2014; Klumpers et al., 2015).

In contrast to previous findings of associations between *5-HTTLPR* and *STin2* VNTR and IMDs (Battersby et al., 1996; Evans et al., 2013; Gressier et al., 2013; Zhao et al., 2013; Smoller, 2016; Wang et al., 2016; Xiao et al., 2019), we found no direct association between any of the investigated *SLC6A4* polymorphisms and IMDs. This may be due to the complex nature of IMDs, where many genetic variants contribute to disease risk, each contributing a small effect (Plomin and Davis, 2009) and thus the effect of a few variants may not be powerful enough to show significant association. Indeed, studies examining the contributions of *5-HTTLPR* and *STin2* variants have yielded inconsistent results, with other published work similarly failing to find a significant association (Munafò et al., 2009a,b; Culverhouse et al., 2018; Xiao et al., 2019). In order to resolve these contradictory results, studies looking at epistatic interactions are required.

Based on the diathesis-stress hypothesis of neuropsychiatric disorders, we postulated that the association between acute stress and IMDs would be moderated by genetic polymorphisms, chronic stress, or a combination of genetic polymorphisms and chronic stress. Results revealed chronic stress and the *5-HTTLPR*-rs25531 *S-A-S-A* haplotype to each significantly moderate the association between acute stress and IMDs.

The interaction between the *5-HTTLPR* and stress has previously been investigated in gene-environment studies of IMDs (Karg et al., 2011; Sharpley et al., 2014). In contrast to previous studies that have reported the moderating role of *5-HTTLPR* on the association between stress and IMDs (Caspi et al., 2003; Karg et al., 2011; Conway et al., 2014; Sharpley et al., 2014), we found no significant moderating effect of *5-HTTLPR* on the association between acute stress and IMDs. However, our results revealed that the combined *5-HTTLPR*-rs25531 haplotype significantly moderates the association between acute stress and IMDs. These results revealed the *S-A-S-A* genotype as a vulnerability factor for IMDs. HIV+ children who experienced moderate and severe acute stress and possessed the *S-A-S-A* haplotype were more likely to have an IMD, compared to HIV+ children who experienced mild acute stress and possessed the *L-A-L-A* haplotype.

Previous studies have reported the *SS* genotype to be a risk for different IMD psychopathologies including depression (Zalsman et al., 2006; Cervilla et al., 2007; Karg et al., 2011; Sharpley et al., 2014; Haberstick et al., 2016), anxiety (Armbruster et al., 2009), and IMDs in general (Conway et al., 2014). However, we found no association between the *SS* genotype and IMDs in the present study. This observation could be due to insufficient power to detect the effect of *5-HTTLPR* on the association between acute stress and IMDs among our study participants. Our study sample size had a *post hoc* power of greater than 80% (87.5% for the interaction analyses) under assumption that there was zero correlation of exposure between cases and controls and that the probability of exposure among controls was 0.5. There is, however, a possibility that the probability of exposure could have been less than 0.5 among controls, thus reducing the power in the present study. The association between the *SS* genotype and IMDs has been suggested to be due to its lower transcriptional activity (Baca-García et al., 2002; Hu et al., 2006). Future functional studies that examine the effects of the *S-A-S-A* compared to the *L-A-L-A* haplotype are required for us to determine whether lower 5-HTT levels could be driving our results.

A significant association was observed between *TPH2* rs1843809 and IMDs. The functional role of *TPH2* rs1843809 is currently not known. Being an intronic variant, it is less likely that rs1843809 would have an effect on gene expression or protein structure, as introns are spliced during mRNA processing, although potential effects of intronic variants have been suggested (Kleinjan and van Heyningen, 2005). It is also possible that rs1843809 is in LD with a causal variant. For example, rs1843809 is in LD with a missense rs142055199 SNP among the Luhya, a population that speaks the same Niger-Congo language as the population of the present study (Baganda; Countries and their Cultures, 2021). East African populations that speak this class of language have been found to be genetically similar by principal components analysis on GWAS data from NeuroGAP pilot study (Unpublished results). The SNP rs142055199 is located in the zinc finger C3H1-type containing gene, which modulates interleukin-8 (IL-8) transcription (National Center for Biotechnology Information, 2021c). Modulation of IL-8 would be of interest since IMDs have been associated with inflammatory processes (Miller et al., 2009; Musinguzi et al., 2018; Hori and Kim, 2019; Wang et al., 2019). Further studies will be needed to determine the mechanisms underlying the influence of this SNP in IMDs.

None of the investigated polymorphisms in *TPH2* moderated the association between acute stress and IMDs. This suggests that although TPH2 is critical in 5-HT biosynthesis (Walther et al., 2003; Carkaci-Salli et al., 2006), genetic variants investigated in this study neither moderate an individual’s response to stress nor are they causal polymorphisms that moderate an individual’s response to stress.

### Limitations and Recommendations

The following limitations should be noted. We defined IMDs as having any depressive disorder, anxiety disorder, or PTSD. As much as anxiety disorders are commonly comorbid with depression, the inclusion of PTSD (*n* = 60) is contentious as it has been excluded from the anxiety disorder category in the Diagnostic and Statistical Manual of Mental Disorders – 5th edition (DSM-5) and is not widely accepted as an IMD (American Psychiatric Association, 2013). The inclusion of PTSD samples allowed us to achieve the desired statistical power. However, the low correlation between genetic risk factors for PTSD and those of depression and anxiety would have reduced the power to detect true significant associations and interactions. Future studies should endeavor to stratify analyses by specific IMDs although this will require larger samples. In addition, both acute and chronic stress indices were measured using a number of context-specific indicators. This approach was adopted because there is no locally adapted tool for assessing these indicators in this setting and the variables used to generate these indices are known stressors in this population. However, this tool remains to be validated. Moreover, we did not control for population stratification at analysis. The study participants belong to the Bagandan population group, for which principal components analysis on a GWAS sample of over 4,700 individuals has shown less genetic variation among the Bagandan (unpublished data). There is, however, a possibility that some participants were not Bagandan, even though they could speak Luganda (the main language of Baganda), indicating that we may have had an admixed sampled. Future studies should, therefore, control for population stratification, in order to circumvent confounding of results due to possible ancestry associated differences in genetic architecture. Also, due to the exploratory nature of the study, multiple testing corrections were not done. Correcting for multiple testing would have rendered the interaction analysis non-significant. We, therefore, acknowledge the risk of false positive findings in this study which have potentially important clinical implications and need to be verified in future research.

## Conclusion

Acute stress was found to be associated with an increased risk of IMDs, and this association was found to be moderated by the 5-*HTTLPR*-rs25531 *S-A-S-A* haplotype and chronic stress among Ugandan HIV+ children and adolescents. These results support the diathesis-stress model, though the mechanisms through which acute stress interacts with *5-HTTLPR*-rs25531 *S-A-S-A* haplotype to moderate the risk of IMDs need to be elucidated. This will allow for interventions to be targeted to at-risk individuals, an important consideration in resource constrained settings.

## Data Availability Statement

All information gathered about study subjects and their samples is confidential, with access limited to the research team. However, upon request, data from the MRC/UVRI and LSHTM Uganda Research Unit is currently accessed under a data sharing policy *via*: http://www.mrcuganda.org/sites/default/files/publications/MRC_UVRI_Data_sharing_policy_December2015.pdf.

## Ethics Statement

This study was reviewed and approved by the Health Research Committee of Stellenbosch University (# S17/09/179) and the higher Degrees Research and Ethics Committee, School of Biomedical Sciences, College of Health Sciences, Makerere University (# SBS 421). The parent study (CHAKA) obtained ethics approval from the Uganda Virus Research Institute (UVRI) Science and Ethical Committee (# GC/127/15/06/459) and the Uganda National Council of Science and Technology (# HS 1601). All parents/caregivers provided written informed consent for their children/adolescents to participate in the study and for a blood specimen to be withdrawn from them for the genetics analyses. Adolescents further provided written informed assent to participate in the study. Written informed consent to participate in this study was provided by the participants’ legal guardian/next of kin.

## Author Contributions

AK, SH, EK, and SS: concept. AK, EK, SH, SS, and JW: data collection. WS, AK, RN, SH, JW, SS, and EK: data analysis. AK, SH, SS, EK, JW, MJ, WS, and RN: First draft and final revision. All authors contributed to the article and approved the submitted version.

### Conflict of Interest

The authors declare that the research was conducted in the absence of any commercial or financial relationships that could be construed as a potential conflict of interest.

The handling editor declared a past co-authorship with the author RN.

## References

[ref1] Adelman R. D. Tmanova L. L. Delgado D. Dion S. Lachs M. S. (2014). Caregiver burden: a clinical review. JAMA 311, 1052–1060. 10.1001/jama.2014.304, PMID: 24618967

[ref2] Ali F. R. Vasiliou S. A. Haddley K. Paredes U. M. Roberts J. C. Miyajima F.. (2010). Combinatorial interaction between two human serotonin transporter gene variable number tandem repeats and their regulation by CTCF. J. Neurochem. 112, 296–306. 10.1111/j.1471-4159.2009.06453.x, PMID: 19860858 PMC2848977

[ref29] American Psychiatric Association (2013). Diagnostic and Statistical Manual of Mental Disorders. 5th Edn.

[ref3] Anttila S. Viikki M. Huuhka K. Huuhka M. Huhtala H. Rontu R.. (2009). TPH2 polymorphisms may modify clinical picture in treatment-resistant depression. Neurosci. Lett. 464, 43–46. 10.1016/j.neulet.2009.08.018, PMID: 19679166

[ref4] Armbruster D. Moser D. A. Strobel A. Hensch T. Kirschbaum C. Lesch K. P.. (2009). Serotonin transporter gene variation and stressful life events impact processing of fear and anxiety. Int. J. Neuropsychopharmacol. 12, 393–401. 10.1017/S1461145708009565, PMID: 18925984

[ref5] Artigas F. (2013a). Serotonin receptors involved in antidepressant effects. Pharmacol. Ther. 137, 119–131. 10.1016/j.pharmthera.2012.09.006 23022360

[ref6] Artigas F. (2013b). Future directions for serotonin and antidepressants. ACS Chem. Neurosci. 4, 5–8. 10.1021/cn3001125 23336036 PMC3547492

[ref7] Assary E. Vincent J. P. Keers R. Pluess M. (2018). Gene-environment interaction and psychiatric disorders: review and future directions. Semin. Cell Dev. Biol. 77, 133–143. 10.1016/j.semcdb.2017.10.016, PMID: 29051054

[ref8] Baca-García E. Vaquero C. Diaz-Sastre C. Saiz-Ruiz J. Fernández-Piqueras J. de Leon J. (2002). A gender-specific association between the serotonin transporter gene and suicide attempts. Neuropsychopharmacology 26, 692–695. 10.1016/S0893-133X(01)00394-3, PMID: 11927194

[ref9] Barrett J. C. (2009). Haploview: visualization and analysis of SNP genotype data. Cold Spring Harb. Protoc. 2009:pdb.ip71. 10.1101/pdb.ip71, PMID: 20147036

[ref10] Battersby S. Ogilvie A. D. Smith C. A. Blackwood D. H. Muir W. J. Quinn J. P.. (1996). Structure of a variable number tandem repeat of the serotonin transporter gene and association with affective disorder. Psychiatr. Genet. 6, 177–181. 10.1097/00041444-199624000-00001, PMID: 9149321

[ref11] Betancourt T. Scorza P. Kanyanganzi F. Fawzi M. C. Sezibera V. Cyamatare F.. (2014). HIV and child mental health: a case-control study in Rwanda. Pediatrics 134, e464–e472. 10.1542/peds.2013-2734, PMID: 25049342 PMC4187226

[ref12] Beusenberg M. Orley J. H. (1994). A User's Guide to The Self Reporting Questionnaire (SRQ). Geneva, Switzerland: World Health Organization.

[ref13] Bhatia R. Hartman C. Kallen M. A. Graham J. Giordano T. P. (2011). Persons newly diagnosed with HIV infection are at high risk for depression and poor linkage to care: results from the steps study. AIDS Behav. 15, 1161–1170. 10.1007/s10461-010-9778-9, PMID: 20711651 PMC3029485

[ref14] Boyes M. E. Cluver L. D. (2015). Relationships between familial HIV/AIDS and symptoms of anxiety and depression: the mediating effect of bullying victimization in a prospective sample of south African children and adolescents. J. Youth Adolesc. 44, 847–859. 10.1007/s10964-014-0146-3, PMID: 24996836

[ref15] Brown R. C. Nugent N. R. Hawn S. E. Koenen K. C. Miller A. Amstadter A. B.. (2016). Predicting the transition from acute stress disorder to posttraumatic stress disorder in children with severe injuries. J. Pediatr. Health Care 30, 558–568. 10.1016/j.pedhc.2015.11.015, PMID: 26776839 PMC4945483

[ref16] Carkaci-Salli N. Flanagan J. M. Martz M. K. Salli U. Walther D. J. Bader M.. (2006). Functional domains of human tryptophan hydroxylase 2 (hTPH2). J. Biol. Chem. 281, 28105–28112. 10.1074/jbc.M602817200 16864580

[ref17] Caspi A. Moffitt T. E. (2006). Gene-environment interactions in psychiatry: joining forces with neuroscience. Nat. Rev. Neurosci. 7, 583–590. 10.1038/nrn1925, PMID: 16791147

[ref18] Caspi A. Sugden K. Moffitt T. E. Taylor A. Craig I. W. Harrington H.. (2003). Influence of life stress on depression: moderation by a polymorphism in the 5-HTT gene. Science 301, 386–389. 10.1126/science.1083968, PMID: 12869766

[ref19] Cervilla J. A. Molina E. Rivera M. Torres-González F. Bellón J. A. Moreno B.. (2007). The risk for depression conferred by stressful life events is modified by variation at the serotonin transporter 5HTTLPR genotype: evidence from the Spanish PREDICT-gene cohort. Mol. Psychiatry 12, 748–755. 10.1038/sj.mp.4001981, PMID: 17387319

[ref20] Charney D. S. Manji H. K. (2004). Life stress, genes, and depression: multiple pathways lead to increased risk and new opportunities for intervention. Sci. STKE 2004, re5. 10.1126/stke.2252004re5, PMID: 15039492

[ref21] Chida Y. Vedhara K. (2009). Adverse psychosocial factors predict poorer prognosis in HIV disease: a meta-analytic review of prospective investigations. Brain Behav. Immun. 23, 434–445. 10.1016/j.bbi.2009.01.013 19486650

[ref22] Cipriani A. Furukawa T. A. Salanti G. Chaimani A. Atkinson L. Z. Ogawa Y.. (2018). Comparative efficacy and acceptability of 21 antidepressant drugs for the acute treatment of adults with major depressive disorder: a systematic review and network meta-analysis. Lancet 391, 1357–1366. 10.1016/S0140-6736(17)32802-7, PMID: 29477251 PMC5889788

[ref1800] Clarke H. Flint J. Attwood A. S. Munafò M. R. (2010). Association of the 5- HTTLPR genotype and unipolar depression: a meta-analysis. Psychol Med. 40, 1767–1778. 10.1017/S0033291710000516, PMID: 20380781

[ref23] Clarke L. Fairley S. Zheng-Bradley X. Streeter I. Perry E. Lowy E.. (2017). The international genome sample resource (IGSR): a worldwide collection of genome variation incorporating the 1000 genomes project data. Nucleic Acids Res. 45, D854–D859. 10.1093/nar/gkw829, PMID: 27638885 PMC5210610

[ref24] Conway C. C. Slavich G. M. Hammen C. (2014). Daily stress reactivity and serotonin transporter gene (5-HTTLPR) variation: internalizing responses to everyday stress as a possible transdiagnostic phenotype. Biol. Mood Anxiety Disord. 4:2. 10.1186/2045-5380-4-2, PMID: 24461074 PMC3933324

[ref25] Countries and their Cultures (2021). Baganda. [Place unknown]. [Publisher unknown]. [Date unknown, cited 2021 Jan 02]. Available at: https://www.everyculture.com/wc/Tajikistan-to-Zimbabwe/Baganda.html (Accessed January 02, 2020).

[ref26] Culverhouse R. C. Saccone N. L. Horton A. C. Ma Y. Anstey K. J. Banaschewski T.. (2018). Collaborative meta-analysis finds no evidence of a strong interaction between stress and 5-HTTLPR genotype contributing to the development of depression. Mol. Psychiatry 23, 133–142. 10.1038/mp.2017.44, PMID: 28373689 PMC5628077

[ref27] Davies M. N. Verdi S. Burri A. Trzaskowski M. Lee M. Hettema J. M.. (2015). Generalised anxiety disorder--a twin study of genetic architecture, genome-wide association and differential gene expression. PLoS One 10:e0134865. 10.1371/journal.pone.0134865, PMID: 26274327 PMC4537268

[ref28] de Kloet E. R. Joëls M. Holsboer F. (2005). Stress and the brain: from adaptation to disease. Nat. Rev. Neurosci. 6, 463–475. 10.1038/nrn1683, PMID: 15891777

[ref30] Ehli E. A. Hu Y. Lengyel-Nelson T. Hudziak J. J. Davies G. E. (2012). Identification and functional characterization of three novel alleles for the serotonin transporter-linked polymorphic region. Mol. Psychiatry 17, 185–192. 10.1038/mp.2010.130, PMID: 21200389

[ref31] Evans G. W. Li D. Whipple S. S. (2013). Cumulative risk and child development. Psychol. Bull. 139, 1342–1396. 10.1037/a0031808, PMID: 23566018

[ref32] Fuchs E. Flïugge G. (2006). Experimental animal models for the simulation of depression and anxiety. Dialogues Clin. Neurosci. 8, 323–333. PMID: 17117614 10.31887/DCNS.2006.8.3/efuchs PMC3181820

[ref33] Fullerton C. S. Ursano R. J. Wang L. (2004). Acute stress disorder, posttraumatic stress disorder, and depression in disaster or rescue workers. Am. J. Psychiatry 161, 1370–1376. 10.1176/appi.ajp.161.8.1370, PMID: 15285961

[ref34] Furlong R. A. Ho L. Walsh C. Rubinsztein J. S. Jain S. Paykel E. S.. (1998). Analysis and meta-analysis of two serotonin transporter gene polymorphisms in bipolar and unipolar affective disorders. Am. J. Med. Genet. 81, 58–63. 10.1002/(SICI)1096-8628(19980207)81:1<58::AID-AJMG11>3.0.CO;2-V, PMID: 9514589

[ref350] Gadow K. D. Sprafkin J. (1999). Youth’s Inventory-4. Stony Brook, New York: Checkmate plus; [Revised 2016 Jan 23]. Available at: https://www.checkmateplus.com/product/yi-4.htm (Accessed April 07, 2021).

[ref35] Gadow K. D. Sprafkin J. (2013). Child and Adolescent Symptom Inventory-5. Stony Brook, New York: Checkmate plus; [Revised 2016 Jan 23]. Available at: https://www.checkmateplus.com/product/casi5.htm (Accessed April 07, 2021).

[ref36] Gao J. Pan Z. Jiao Z. Li F. Zhao G. Wei Q.. (2012). TPH2 gene polymorphisms and major depression--a meta-analysis. PLoS One 7:e36721. 10.1371/journal.pone.0036721, PMID: 22693556 PMC3365065

[ref37] Gressier F. Calati R. Balestri M. Marsano A. Alberti S. Antypa N.. (2013). The 5-HTTLPR polymorphism and posttraumatic stress disorder: a meta-analysis. J. Trauma. Stress. 26, 645–653. 10.1002/jts.21855, PMID: 24222274

[ref38] Grillon C. Duncko R. Covington M. F. Kopperman L. Kling M. A. (2007). Acute stress potentiates anxiety in humans. Biol. Psychiatry 62, 1183–1186. 10.1016/j.biopsych.2007.06.007, PMID: 17692829 PMC2093988

[ref39] Gurdasani D. Carstensen T. Tekola-Ayele F. Pagani L. Tachmazidou I. Hatzikotoulas K.. (2015). The African genome variation project shapes medical genetics in Africa. Nature 517, 327–332. 10.1038/nature13997, PMID: 25470054 PMC4297536

[ref40] Haberstick B. C. Boardman J. D. Wagner B. Smolen A. Hewitt J. K. Killeya-Jones L. A.. (2016). Depression, stressful life events, and the impact of variation in the serotonin transporter: findings from the National Longitudinal Study of adolescent to adult health (add health). PLoS One 11:e0148373. 10.1371/journal.pone.0148373, PMID: 26938215 PMC4777542

[ref41] Haddley K. Bubb V. J. Breen G. Parades-Esquivel U. M. Quinn J. P. (2012). Behavioural genetics of the serotonin transporter. Curr. Top. Behav. Neurosci. 12, 503–535. 10.1007/7854_2011_186 22261701

[ref42] Hammoud D. A. Endres C. J. Hammond E. Uzuner O. Brown A. Nath A.. (2010). Imaging serotonergic transmission with [11C]DASB-PET in depressed and non-depressed patients infected with HIV. NeuroImage 49, 2588–2595. 10.1016/j.neuroimage.2009.10.037, PMID: 19853044 PMC2818313

[ref43] Heils A. Teufel A. Petri S. Stöber G. Riederer P. Bengel D.. (1996). Allelic variation of human serotonin transporter gene expression. J. Neurochem. 66, 2621–2624. 10.1046/j.1471-4159.1996.66062621.x, PMID: 8632190

[ref44] Hettema J. M. Neale M. C. Kendler K. S. (2001). A review and meta-analysis of the genetic epidemiology of anxiety disorders. Am. J. Psychiatry 158, 1568–1578. 10.1176/appi.ajp.158.10.1568, PMID: 11578982

[ref45] Hori H. Kim Y. (2019). Inflammation and post-traumatic stress disorder. Psychiatry Clin. Neurosci. 73, 143–153. 10.1111/pcn.12820, PMID: 30653780

[ref46] Hu X. Z. Lipsky R. H. Zhu G. Akhtar L. A. Taubman J. Greenberg B. D.. (2006). Serotonin transporter promoter gain-of-function genotypes are linked to obsessive-compulsive disorder. Am. J. Hum. Genet. 78, 815–826. 10.1086/503850, PMID: 16642437 PMC1474042

[ref47] Ironson G. O'Cleirigh C. Fletcher M. A. Laurenceau J. P. Balbin E. Klimas N.. (2005). Psychosocial factors predict CD4 and viral load change in men and women with human immunodeficiency virus in the era of highly active antiretroviral treatment. Psychosom. Med. 67, 1013–1021. 10.1097/01.psy.0000188569.58998.c8, PMID: 16314608 PMC2614887

[ref48] Jacob J. Ferri A. L. Milton C. Prin F. Pla P. Lin W.. (2007). Transcriptional repression coordinates the temporal switch from motor to serotonergic neurogenesis. Nat. Neurosci. 10, 1433–1439. 10.1038/nn1985, PMID: 17922007

[ref49] Jawahar M. C. Toben C. G. Baune B. T. (2019). “Gene-environment interactions and epigenetic mechanisms in depression” in Neurobiology of Depression. eds. Quevedo J. Carvalho A. F. Zarate C. A. (Academic Press), 17–25.

[ref50] Kalungi A. Kinyanda E. Womersley J. S. Joloba M. L. Ssembajjwe W. Nsubuga R. N.. (2021). TERT rs2736100 and TERC rs16847897 genotypes moderate the association between internalizing mental disorders and accelerated telomere length attrition among HIV+ children and adolescents in Uganda. BMC Med. Genet. 14:15. 10.1186/s12920-020-00857-z PMC7789327 33407441

[ref51] Kalungi A. Seedat S. Hemmings S. M. J. van der Merwe L. Joloba M. L. Nanteza A.. (2017). Association between serotonin transporter gene polymorphisms and increased suicidal risk among HIV positive patients in Uganda. BMC Genet. 18:71. 10.1186/s12863-017-0538-y, PMID: 28743254 PMC5526289

[ref52] Kalungi A. Womersley J. S. Kinyanda E. Joloba M. L. Ssembajjwe W. Nsubuga R. N.. (2019). Internalizing mental disorders and accelerated cellular aging among perinatally HIV-infected youth in Uganda. Front. Genet. 10:705. 10.3389/fgene.2019.00705 31428136 PMC6688656

[ref53] Karg K. Burmeister M. Shedden K. Sen S. (2011). The serotonin transporter promoter variant (5-HTTLPR), stress, and depression meta-analysis revisited: evidence of genetic moderation. Arch. Gen. Psychiatry 68, 444–454. 10.1001/archgenpsychiatry.2010.189, PMID: 21199959 PMC3740203

[ref54] Kennedy A. P. Binder E. B. Bowman D. Harenski K. Ely T. Cisler J. M.. (2012). A common TPH2 haplotype regulates the neural processing of a cognitive control demand. Am. J. Med. Genet. B Neuropsychiatr. Genet. 159B, 829–840. 10.1002/ajmg.b.32090, PMID: 22915309

[ref55] Kinyanda E. Levin J. Nakasujja N. Birabwa H. Nakku J. Mpango R.. (2018). Major depressive disorder: longitudinal analysis of impact on clinical and behavioral outcomes in Uganda. J. Acquir. Immune Defic. Syndr. 78, 136–143. 10.1097/QAI.0000000000001647 29424787

[ref56] Kinyanda E. Salisbury T. T. Levin J. Nakasujja N. Mpango R. S. Abbo C.. (2019). Rates, types and co-occurrence of emotional and behavioural disorders among perinatally HIV-infected youth in Uganda: the CHAKA study. Soc. Psychiatry Psychiatr. Epidemiol. 54, 415–425. 10.1007/s00127-019-01675-0, PMID: 30788554

[ref57] Kleinjan D. A. van Heyningen V. (2005). Long-range control of gene expression: emerging mechanisms and disruption in disease. Am. J. Hum. Genet. 76, 8–32. 10.1086/426833, PMID: 15549674 PMC1196435

[ref58] Klumpers F. Kroes M. C. Heitland I. Everaerd D. Akkermans S. E. Oosting R. S.. (2015). Dorsomedial prefrontal cortex mediates the impact of serotonin transporter linked polymorphic region genotype on anticipatory threat reactions. Biol. Psychiatry 78, 582–589. 10.1016/j.biopsych.2014.07.034, PMID: 25444169

[ref59] Klumpp H. Fitzgerald D. A. Cook E. Shankman S. A. Angstadt M. Phan K. L. (2014). Serotonin transporter gene alters insula activity to threat in social anxiety disorder. Neuroreport 25, 926–931. 10.1097/WNR.0000000000000210, PMID: 25003950 PMC4371777

[ref60] Kristensen A. S. Andersen J. Jørgensen T. N. Sørensen L. Eriksen J. Loland C. J.. (2011). SLC6 neurotransmitter transporters: structure, function, and regulation. Pharmacol. Rev. 63, 585–640. 10.1124/pr.108.000869 21752877

[ref61] Lachin J. M. (1992). Power and sample size evaluation for the McNemar test with application to matched case-control studies. Stat. Med. 11, 1239–1251. 10.1002/sim.4780110909, PMID: 1509223

[ref62] Lesch K. P. Bengel D. Heils A. Sabol S. Z. Greenberg B. D. Petri S.. (1996). Association of anxiety-related traits with a polymorphism in the serotonin transporter gene regulatory region. Science 274, 1527–1531. 10.1126/science.274.5292.1527, PMID: 8929413

[ref63] Lipsky R. H. Hu X. Z. Goldman D. (2009). Additional functional variation at the SLC6A4 gene. Am. J. Med. Genet. B Neuropsychiatr. Genet. 150B:153. 10.1002/ajmg.b.30766, PMID: 18444253 PMC2716041

[ref64] Lopez de Lara C. Brezo J. Rouleau G. Lesage A. Dumont M. Alda M.. (2007). Effect of tryptophan hydroxylase-2 gene variants on suicide risk in major depression. Biol. Psychiatry 62, 72–80. 10.1016/j.biopsych.2006.09.008, PMID: 17217922

[ref65] Lwidiko A. Kibusi S. M. Nyundo A. Mpondo B. C. T. (2018). Association between HIV status and depressive symptoms among children and adolescents in the southern highlands zone, Tanzania: a case-control study. PLoS One 13:e0193145. 10.1371/journal.pone.0193145, PMID: 29470512 PMC5823441

[ref66] Martin J. Cleak J. Willis-Owen S. A. Flint J. Shifman S. (2007). Mapping regulatory variants for the serotonin transporter gene based on allelic expression imbalance. Mol. Psychiatry 12, 421–422. 10.1038/sj.mp.4001952, PMID: 17453058

[ref67] Mellins C. A. Elkington K. S. Leu C. S. Santamaria E. K. Dolezal C. Wiznia A.. (2012). Prevalence and change in psychiatric disorders among perinatally HIV-infected and HIV-exposed youth. AIDS Care 24, 953–962. 10.1080/09540121.2012.668174, PMID: 22519762 PMC3416047

[ref68] Miller A. H. Maletic V. Raison C. L. (2009). Inflammation and its discontents: the role of cytokines in the pathophysiology of major depression. Biol. Psychiatry 65, 732–741. 10.1016/j.biopsych.2008.11.029, PMID: 19150053 PMC2680424

[ref69] Monroe S. M. Simons A. D. (1991). Diathesis-stress theories in the context of life stress research: implications for the depressive disorders. Psychol. Bull. 110, 406–425. 10.1037/0033-2909.110.3.406, PMID: 1758917

[ref70] Mpango R. S. Kinyanda E. Rukundo G. Z. Gadow K. D. Patel V. (2017). Cross-cultural adaptation of the child and adolescent symptom Inventory-5 (CASI-5) for use in central and South-Western Uganda: the CHAKA project. Trop. Dr. 47, 347–354. 10.1177/0049475517724688 28766999

[ref71] Munafò M. R. Durrant C. Lewis G. Flint J. (2009a). Gene X environment interactions at the serotonin transporter locus. Biol. Psychiatry 65, 211–219. 10.1016/j.biopsych.2008.06.009 18691701

[ref72] Munafò M. R. Freimer N. B. Ng W. Ophoff R. Veijola J. Miettunen J.. (2009b). 5-HTTLPR genotype and anxiety-related personality traits: a meta-analysis and new data. Am. J. Med. Genet. B Neuropsychiatr. Genet. 150B, 271–281. 10.1002/ajmg.b.30808 18546120 PMC2819421

[ref73] Musci R. J. Augustinavicius J. L. Volk H. (2019). Gene-environment interactions in psychiatry: recent evidence and clinical implications. Curr. Psychiatry Rep. 21:81. 10.1007/s11920-019-1065-5 31410638 PMC7340157

[ref74] Musinguzi K. Obuku A. Nakasujja N. Birabwa H. Nakku J. Levin J.. (2018). Association between major depressive disorder and pro-inflammatory cytokines and acute phase proteins among HIV-1 positive patients in Uganda. BMC Immunol. 19:1. 10.1186/s12865-017-0239-3, PMID: 29298663 PMC5751544

[ref75] Nachman S. Chernoff M. Williams P. Hodge J. Heston J. Gadow K. D. (2012). Human immunodeficiency virus disease severity, psychiatric symptoms, and functional outcomes in perinatally infected youth. Arch. Pediatr. Adolesc. Med. 166, 528–535. 10.1001/archpediatrics.2011.1785, PMID: 22312169 PMC3407294

[ref76] National Center for Biotechnology Information (2021a). SLC6A4 solute carrier family 6 member 4 [*Homo sapiens* (human)]. Updated 2020 Dec 27. Available at: https://www.ncbi.nlm.nih.gov/gene/6532#location (Accessed December 29, 2020).

[ref77] National Center for Biotechnology Information (2021b). STIN2-VNTR serotonin transporter intronic VNTR enhancer [*Homo sapiens* (human)]. Updated 2020 Nov 24. Available at: https://www.ncbi.nlm.nih.gov/gene/?term=serotonin+transporter+intronic+VNTR+enhancer+%5BHomo+sapiens+(human)%5D (Accessed December 29, 2020).

[ref78] National Center for Biotechnology Information (2021c). ZFC3H1 zinc finger C3H1-type containing [*Homo sapiens* (human)]. Updated 2020 Dec 06. Available at: https://www.ncbi.nlm.nih.gov/gene/196441 (Accessed December 29, 2020).

[ref79] O’Connor R. C. Rasmussen S. Hawton K. (2010). Predicting depression, anxiety and self-harm in adolescents: the role of perfectionism and acute life stress. Behav. Res. Ther. 48, 52–59. 10.1016/j.brat.2009.09.008, PMID: 19818954

[ref80] O’Gara C. Knight J. Stapleton J. Luty J. Neale B. Nash M.. (2008). Association of the serotonin transporter gene, neuroticism and smoking behaviours. J. Hum. Genet. 53, 239–246. 10.1007/s10038-007-0243-1, PMID: 18188666

[ref81] Olivier B. (2015). Serotonin: a never-ending story. Eur. J. Pharmacol. 753, 2–18. 10.1016/j.ejphar.2014.10.031, PMID: 25446560

[ref82] Otte C. Gold S. M. Penninx B. W. Pariante C. M. Etkin A. Fava M.. (2016). Major depressive disorder. Nat. Rev. Dis. Primers. 2:16065. 10.1038/nrdp.2016.65 27629598

[ref83] Pergamin-Hight L. Bakermans-Kranenburg M. J. van Ijzendoorn M. H. Bar-Haim Y. (2012). Variations in the promoter region of the serotonin transporter gene and biased attention for emotional information: a meta-analysis. Biol. Psychiatry 71, 373–379. 10.1016/j.biopsych.2011.10.030, PMID: 22138391

[ref84] Philibert R. A. Sandhu H. Hollenbeck N. Gunter T. Adams W. Madan A. (2008). The relationship of 5HTT (SLC6A4) methylation and genotype on mRNA expression and liability to major depression and alcohol dependence in subjects from the Iowa adoption studies. Am. J. Med. Genet. B Neuropsychiatr. Genet. 147B, 543–549. 10.1002/ajmg.b.30657, PMID: 17987668 PMC3643119

[ref85] Plomin R. Davis O. S. (2009). The future of genetics in psychology and psychiatry: microarrays, genome-wide association, and non-coding RNA. J. Child Psychol. Psychiatry 50, 63–71. 10.1111/j.1469-7610.2008.01978.x, PMID: 19220590 PMC2898937

[ref86] Popoli M. Yan Z. McEwen B. S. Sanacora G. (2011). The stressed synapse: the impact of stress and glucocorticoids on glutamate transmission. Nat. Rev. Neurosci. 13, 22–37. 10.1038/nrn3138, PMID: 22127301 PMC3645314

[ref87] Pristerà A. Lin W. Kaufmann A. K. Brimblecombe K. R. Threlfell S. Dodson P. D.. (2015). Transcription factors FOXA1 and FOXA2 maintain dopaminergic neuronal properties and control feeding behavior in adult mice. Proc. Natl. Acad. Sci. U. S. A. 112, E4929–E4938. 10.1073/pnas.1503911112, PMID: 26283356 PMC4568236

[ref88] Revenson T. Griva K. Luszczynska A. Morrison V. Panagopoulou E. Vilchinsky N.. (2016). Caregiving in the Illness Context. London: Palgrave Macmillan.

[ref89] Robles T. F. Slatcher R. B. Trombello J. M. McGinn M. M. (2014). Marital quality and health: a meta-analytic review. Psychol. Bull. 140, 140–187. 10.1037/a0031859, PMID: 23527470 PMC3872512

[ref90] Rudnick G. (2006). Serotonin transporters--structure and function. J. Membr. Biol. 213, 101–110. 10.1007/s00232-006-0878-4, PMID: 17417703

[ref91] Sharpley C. F. Palanisamy S. K. Glyde N. S. Dillingham P. W. Agnew L. L. (2014). An update on the interaction between the serotonin transporter promoter variant (5-HTTLPR), stress and depression, plus an exploration of non-confirming findings. Behav. Brain Res. 273, 89–105. 10.1016/j.bbr.2014.07.030, PMID: 25078292

[ref92] Shishkina G. T. Kalinina T. S. Dygalo N. N. (2007). Up-regulation of tryptophan hydroxylase-2 mRNA in the rat brain by chronic fluoxetine treatment correlates with its antidepressant effect. Neuroscience 150, 404–412. 10.1016/j.neuroscience.2007.09.017, PMID: 17950541

[ref93] Silberg J. L. Rutter M. Eaves L. (2001). Genetic and environmental influences on the temporal association between earlier anxiety and later depression in girls. Biol. Psychiatry 49, 1040–1049. 10.1016/S0006-3223(01)01161-1, PMID: 11430845

[ref94] Smoller J. W. (2016). The genetics of stress-related disorders: PTSD, depression, and anxiety disorders. Neuropsychopharmacology 41, 297–319. 10.1038/npp.2015.266, PMID: 26321314 PMC4677147

[ref95] Springer S. A. Dushaj A. Azar M. M. (2012). The impact of DSM-IV mental disorders on adherence to combination antiretroviral therapy among adult persons living with HIV/AIDS: a systematic review. AIDS Behav. 16, 2119–2143. 10.1007/s10461-012-0212-3 22644066 PMC3481055

[ref96] Tandon M. Si X. Luby J. (2011). Preschool onset attention-deficit/hyperactivity disorder: course and predictors of stability over 24 months. J. Child Adolesc. Psychopharmacol. 21, 321–330. 10.1089/cap.2010.0045, PMID: 21851190 PMC3157747

[ref97] van den Buuse M. Hale M. W. (2019). “Serotonin in stress” in Stress: Physiology, Biochemistry, and Pathology. ed. Fink G. (Academic Press), 115–123.

[ref98] Walther D. J. Peter J. U. Bashammakh S. Hörtnagl H. Voits M. Fink H.. (2003). Synthesis of serotonin by a second tryptophan hydroxylase isoform. Science 299:76. 10.1126/science.1078197, PMID: 12511643

[ref99] Wang Y. Sun N. Liu Z. Li X. Yang C. Zhang K. (2016). Psychosocial mechanisms of serotonin transporter's genetic polymorphism in susceptibility to major depressive disorder: mediated by trait coping styles and interacted with life events. Am. J. Transl. Res. 8, 1281–1292. PMID: 27158415 PMC4846972

[ref100] Wang W. Wang L. Xu H. Cao C. Liu P. Luo S.. (2019). Characteristics of pro- and anti-inflammatory cytokines alteration in PTSD patients exposed to a deadly earthquake. J. Affect. Disord. 248, 52–58. 10.1016/j.jad.2019.01.029, PMID: 30711869

[ref101] Ward J. J. H. (1963). Hierarchical grouping to optimize an objective function. J. Am. Stat. Assoc. 58, 236–244.

[ref102] Widaman K. F. Helm J. L. Castro-Schilo L. Pluess M. Stallings M. C. Belsky J. (2012). Distinguishing ordinal and disordinal interactions. Psychol. Methods 17, 615–622. 10.1037/a0030003, PMID: 22984788 PMC3553243

[ref103] Willis A. M. Brock M. S. (2018) Adverse Childhood Experience and Serotonin Transporters: A Gene Environmental Study of the Risk of PTSD in Soldiers (ACES). 59 MDW San Antonio United States.

[ref104] Xiao Y. Liu D. Liu K. Wu C. Zhang H. Niu Y.. (2019). Association of DRD2, 5-HTTLPR, and 5-HTTVNTR gene polymorphisms with posttraumatic stress disorder in Tibetan adolescents: a case-control study. Biol. Res. Nurs. 21, 286–295. 10.1177/1099800419838325, PMID: 30983408

[ref105] Xie P. Kranzler H. R. Poling J. Stein M. B. Anton R. F. Brady K.. (2009). Interactive effect of stressful life events and the serotonin transporter 5-HTTLPR genotype on posttraumatic stress disorder diagnosis in 2 independent populations. Arch. Gen. Psychiatry 66, 1201–1209. 10.1001/archgenpsychiatry.2009.153, PMID: 19884608 PMC2867334

[ref106] Zalsman G. Huang Y. Y. Oquendo M. A. Burke A. K. Hu X. Z. Brent D. A.. (2006). Association of a triallelic serotonin transporter gene promoter region (5-HTTLPR) polymorphism with stressful life events and severity of depression. Am. J. Psychiatry 163, 1588–1593. 10.1176/ajp.2006.163.9.1588, PMID: 16946185

[ref107] Zhao J. Miao K. Wang H. Ding H. Wang D. W. (2013). Association between telomere length and type 2 diabetes mellitus: a meta-analysis. PLoS One 8:e79993. 10.1371/journal.pone.0079993, PMID: 24278229 PMC3836967

[ref108] Zill P. Baghai T. C. Zwanzger P. Schüle C. Eser D. Rupprecht R.. (2004). SNP and haplotype analysis of a novel tryptophan hydroxylase isoform (TPH2) gene provide evidence for association with major depression. Mol. Psychiatry 9, 1030–1036. 10.1038/sj.mp.4001525, PMID: 15124006

