# Supplementary material for: The 5-HTTLPR-rs25531 S-A-S-A Haplotype and Chronic Stress Moderate the Association Between Acute Stress and Internalizing Mental Disorders Among HIV+ Children and Adolescents in Uganda
Source: Front Genet. 2021 Apr 23;12:649055. doi: 10.3389/fgene.2021.649055 (PMC8104030; doi:10.3389/fgene.2021.649055)
Supplement: Supplementary file 1 [file Data_Sheet_1.docx]

**Supplementary materials**

**Table S1:** Biological data based on for the selection of variants investigated

| **Gene name** | **Symbol** | **Gene ID (NCBI)** | **Variant** | **MAF (Luhya)** | **Location of variant in the gene** | **Function of the Variant** | **Citation** |
| --- | --- | --- | --- | --- | --- | --- | --- |
| Serotonin transporter | 5-HTT | 6532 | rs*25531* | 0.29 | Chr17: 28,564,346 | Regulates gene expression | (Voyiaziakis *et al*., 2011) |
|  |  |  | STin2.VNTRs | 0.27 | Chr5: 1-180,915,260 | Regulates gene expression |  |
| Tryptophan hydroxylase 2 | TPH2 | 121278 | rs*4570625* | 0.35 | Chr12: 72,331,923 | Associated with MDD | (Gao *et al*., 2012) |
|  |  |  | rs*1843809* | 0.40 | Chr12: 72,348,698 | Associated with MDD | (Zill *et al*., 2004) |
|  |  |  | rs*1386494* | 0.23 | Chr12:72,352,543 | Associated with MDD | (Zill *et al*., 2004) |
|  |  |  | rs*34517220* | 0.53 | Chr12:72,395,229 | Associated with reduction in depressive symptoms in patients treated with fluoxetine | (Gassó *et al.*, 2017) |

**Figure S1**

LD plot showing linkage disequilibrium (LD) for *SLC6A4* 5*-HTTLPR*, rs25531 and *STin2* VNTR


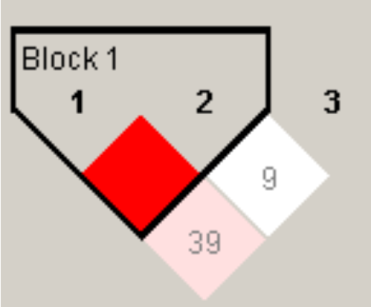


Linkage disequilibrium map of *SLC6A4*. D’ values are depicted in the diamonds with darker colors depicting stronger LD. The LD map was created using the default Gabriel LD (Gabriel *et al*., 2002), implemented in Haploview, version 4.2 (Barrett, 2009). 1 = rs25531, 2 = *5-HTTLPR*, 3 = *STin2* VNTR. *5-HTTLPR* and rs25531 were in LD. Location = 5’ to 3’ on chromosome 17.

**Figure S2**


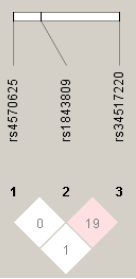


Linkage disequilibrium map of *TPH2*. D’ values are depicted in the diamonds with darker colors depicting stronger LD. The LD map was created using the default Gabriel LD

(Gabriel *et al*., 2002), implemented in Haploview, version 4.2 (Barrett, 2009). 1 = rs4570625, 2 = rs1843809 and 3 = rs34517220. None of the SNPs were in LD. Location = 5’ to 3’ strand on chromosome 12.

**Table S2**: Logistic regression analysis for the interaction of acute stress on selected serotonin transporter gene and tryptophan hydroxylase 2 gene polymorphisms on IMDs

| **Model** | **Variable** | **Odds ratio** | | **P>\|Z\|** | | **95% CI** | **P-value** | |
| --- | --- | --- | --- | --- | --- | --- | --- | --- |
| Excluding any polymorphism | Mild acute stress | Reference | |  | |  | 0.046^ƒ^ | |
|  | Moderate acute stress | 1.22 | | 0.282 | | 0.848 - 1.761 |  |  |
|  | Severe acute stress | 1.92 | | 0.001 | | 1.327 – 2.785 |  |  |
| Including rs1386494 | **Acute stress*rs1386494** | | | | | | | |
|  | Moderate AS**GA* | 1.17 | 0.889 | | 0.125 – 11.004 | | | 0.786^ƒ^ |
|  | Moderate AS**GG* | 1.88 | 0.574 | | 0.208 – 17.067 | | |  |
|  |  |  |  | |  | | |  |
|  | Severe AS**GA* | 0.73 | 0.808 | | 0.056 – 9.508 | | |  |
|  | Severe AS**GG* | 0.94 | 0.963 | | 0.074 – 11.990 | | |  |
| Including rs1843809 | **Acute stress*rs1843809** |  |  | |  | | |  |
|  | Moderate AS**TG* | 1.93 | 0.174 | | 0.750 – 4.951 | | | 0.198^ƒ^ |
|  | Moderate AS**TT* | 3.07 | 0.031 | | 1.110 – 8.490 | | |  |
|  |  |  |  | |  | | |  |
|  | Severe AS**TG* | 0.99 | 0.988 | | 0.359 – 2.743 | | |  |
|  | Severe AS**TT* | 1.89 | 0.253 | | 0.635 – 5.634 | | |  |
| Including rs34517220 | **Acute stress*rs34517220** |  |  | |  | | |  |
|  | Moderate AS**GA* | 1.31 | 0.544 | | 0.544 – 3.177 | | | 0.927^ƒ^ |
|  | Moderate AS**GG* | 1.48 | 0.459 | | 0.527 – 4.138 | | |  |
|  |  |  |  | |  | | |  |
|  | Severe AS**GA* | 1.37 | 0.491 | | 0.557 – 3.378 | | |  |
|  | Severe AS**GG* | 1.23 | 0.685 | | 0.446 – 3.419 | | |  |
| Including rs4570625 | **Acute stress*rs4570625** |  |  | |  | | |  |
|  | Moderate AS**TG* | 2.26 | 0.073 | | 0.926 – 5.532 | | | 0.390^ƒ^ |
|  | Moderate AS**TT* | 2.41 | 0.085 | | 0.886 – 6.568 | | |  |
|  |  |  |  | |  | | |  |
|  | Severe AS**TG* | 1.35 | 0.507 | | 0.554 – 3.299 | | |  |
|  | Severe AS**TT* | 1.21 | 0.719 | | 0.423 – 3.480 | | |  |
| Including rs25531 | **Acute stress*rs25531** |  |  | |  | | |  |
|  | Moderate AS**2* | 1.88 | 0.117 | | 0.853 – 4.153 | | | 0.487^ƒ^ |
|  | Moderate AS*3 | 0.77 | 0.815 | | 0.082 – 7.172 | | |  |
|  |  |  |  | |  | | |  |
|  | Severe AS**2* | 1.46 | 0.362 | | 0.647 – 3.298 | | |  |
|  | Severe AS**3* | 0.43 | 0.404 | | 0.058 – 3.418 | | |  |
| Including *5-HTTLPR* | **Acute stress**5-HTTLPR*** |  |  | |  | | |  |
|  | Moderate AS**LS* | 1.47 | 0.346 | | 0.662 – 3.246 | | | 0.463^ƒ^ |
|  | Moderate AS**SS* | 3.08 | 0.269 | | 0.419 – 22.654 | | |  |
|  |  |  |  | |  | | |  |
|  | Severe AS**LS* | 1.31 | 0.521 | | 0.577 – 2.958 | | |  |
|  | Severe AS**SS* | 4.15 | 0.133 | | 0.647 – 26.574 | | |  |
| Including *STin2.*VNTR | **Acute stress**STin2.*VNTR** |  |  | |  | | |  |
|  | Moderate AS**10/12* | 1.79 | 0.432 | | 0.419 – 7.625 | | | 0.203^ƒ^ |
|  | Moderate AS**12/12* | 1.76 | 0.429 | | 0.434 – 7140 | | |  |
|  |  |  |  | |  | | |  |
|  | Severe AS**10/12* | 0.53 | 0.429 | | 0.106 – 2.592 | | |  |
|  | Severe AS**12/12* | 1.14 | 0.868 | | 0.239 – 5.450 | | |  |

AS = acute stress, * = interaction, ^ƒ^ = p-value for the likelihood-ratio test of interaction between acute stress and the selected polymorphism on IMDs.
